# The Highly Productive *Thermothelomyces heterothallica* C1 Expression System as a Host for Rapid Development of Influenza Vaccines

**DOI:** 10.3390/vaccines10020148

**Published:** 2022-01-20

**Authors:** Gabor Keresztes, Mark Baer, Mark R. Alfenito, Theo C. Verwoerd, Andriy Kovalchuk, Marilyn G. Wiebe, Tor Kristian Andersen, Markku Saloheimo, Ronen Tchelet, Richard Kensinger, Gunnveig Grødeland, Mark Emalfarb

**Affiliations:** 1 Dyadic International Inc., 140 Intracoastal Pointe Drive, Suite 404, Jupiter, FL 33477, USA; gkeresztes@dyadic.com (G.K.); theocverwoerd@hotmail.com (T.C.V.); rtchelet@dyadic.com (R.T.); 2 EnGen Bio LLC, 61 Avondale Ave., Redwood City, CA 94062, USA; mbaer@engenbio.com (M.B.); malfenito@engenbio.com (M.R.A.); 3 VTT Technical Research Centre of Finland Ltd., P.O. Box 1000, 02044 Espoo, Finland; Andriy.Kovalchuk@vtt.fi (A.K.); Marilyn.Wiebe@vtt.fi (M.G.W.); Markku.Saloheimo@vtt.fi (M.S.); 4 Institute of Clinical Medicine, University of Oslo, 0027 Oslo, Norway; t.k.andersen@medisin.uio.no (T.K.A.); gunnveig.grodeland@medisin.uio.no (G.G.); 5 Sanofi Pasteur, 1541 Ave. Marcel Mérieux, 69280 Marcy l’Etoile, France; richard.kensinger@affinivax.com; 6 Department of Immunology and Transfusion Medicine, Oslo University Hospital, 0027 Oslo, Norway

**Keywords:** *Thermothelomyces heterothallica* C1, recombinant protein expression, influenza vaccine, filamentous fungi, trimeric influenza hemagglutinin, targeted influenza hemagglutinin

## Abstract

(1) Influenza viruses constantly change and evade prior immune responses, forcing seasonal re-vaccinations with updated vaccines. Current FDA-approved vaccine manufacturing technologies are too slow and/or expensive to quickly adapt to mid-season changes in the virus or to the emergence of pandemic strains. Therefore, cost-effective vaccine technologies that can quickly adapt to newly emerged strains are desirable. (2) The filamentous fungal host *Thermothelomyces heterothallica* C1 (C1, formerly *Myceliophthora thermophila*) offers a highly efficient and cost-effective alternative to reliably produce immunogens of vaccine quality at large scale. (3) We showed the utility of the C1 system expressing hemagglutinin (HA) and a HA fusion protein from different H1N1 influenza A virus strains. Mice vaccinated with the C1-derived HA proteins elicited anti-HA immune responses similar, or stronger than mice vaccinated with HA products derived from prototypical expression systems. A challenge study demonstrated that vaccinated mice were protected against the aggressive homologous viral challenge. (4) The C1 expression system is proposed as part of a set of protein expression systems for plug-and-play vaccine manufacturing platforms. Upon the emergence of pathogens of concern these platforms could serve as a quick solution for producing enough vaccines for immunizing the world population in a much shorter time and more affordably than is possible with current platforms.

## 1. Introduction

Influenza virus infections account for 3–5 million cases of severe respiratory disease worldwide, associated with 300,000 to 600,000 deaths annually [1,2]. Influenza viruses are enveloped viruses with single stranded RNA genomes of negative polarity (−ssRNA) in the family Orthomyxoviridae. Seasonal influenza virus infections are caused by type A and B influenza viruses (IAV and IBV, respectively), both containing eight −ssRNA segments encoding at least 10–12 ORFs. IAVs are further divided into subtypes based on the antigenic profile of the surface glycoproteins hemagglutinin (HA) and neuraminidase (NA) [3]. There are 18 HA subtypes and 11 NA subtypes, but only H1N1 and H3N2 subtype IAV strains currently circulate in humans [4,5].

Due to the segmented nature of their genomes, influenza viruses can easily re-assort and generate novel strains [6,7]. Pandemic influenza strains are IAVs with HA segments derived from strains in the animal reservoir [8,9] and for which humans carry little to non-existent immunity due to the divergent immunological properties of the HA and NA subtypes. The process of emergence of a novel HA in pandemic IAVs is known as antigenic shift. In contrast, humans are considered the only natural reservoir of IBVs, but have diverged into two antigenically distinct lineages known as Victoria and Yamagata lineages [10]. The HA of both IAV and IBV strains are produced as immature HA0 products that are cleaved by host proteases to generate the globular HA1 and stem HA2 subunits connected by di-sulfide bonds [11]. On the virus, the HA is present as homotrimers stabilized by coiled–coil interactions among the monomers [12]. The HA1 region contains the receptor binding site [13,14] and is the major target of the neutralizing immune responses against influenza [15].

Vaccination is considered the first line of defense against seasonal and pandemic influenza [16]. The HA can easily mutate, particularly on the HA globular HA1 head through the accumulation of small amino acid changes, a process known as antigenic drift [17]. Both antigenic drift and shift makes vaccines largely ineffective after a single season or against newly emerged pandemic strains. Influenza virus vaccines approved for human use trivalent or quadrivalent vaccine formulations against the prevalent H1N1, H3N2, and one or both IBV lineages [18,19]. These vaccines include split virion or subunit inactivated influenza virus (IIV), live attenuated influenza virus (LAIV) vaccines, and recombinant influenza protein (RIV). IIV and LAIV production rely mostly on the use of embryonated chicken eggs and more recently tissue culture cells [20], while RIV are produced by recombinant baculoviruses expressing HA proteins of interest [21]. Despite many advances in terms of vaccine manufacturing and production, these technologies face significant shortcomings about availability and/or efficacy [22].

As has convincingly been demonstrated during the present SARS-CoV-2 pandemic [23,24], an efficient response to an emerging pandemic has been the use of subunit vaccines based on mRNA-nanoparticles [25,26,27] and/or recombinant adenovirus-based vaccines [28]. These approaches are not without drawbacks as stability, reactogenicity, immunogenicity, and production costs are identified as areas for improvement.

To address many of these shortcomings alternative production platforms are needed. Towards that goal, we developed the protein expression system based on the novel filamentous fungal *Thermothelomyces heterothallica* C1. The C1-based system can be used to rapidly develop stable strains in a matter of weeks expressing immunologically active antigens of choice at high yields (compared to other well-established systems). The C1-based system is easily scalable to small and large industrial volumes. It is also highly cost-effective using standard microbial fermentation reactors that are within reach even in areas with limited vaccine production infrastructures.

## 2. Materials and Methods

### 2.1. The Expressed Influenza Antigens

**Figure 1:**
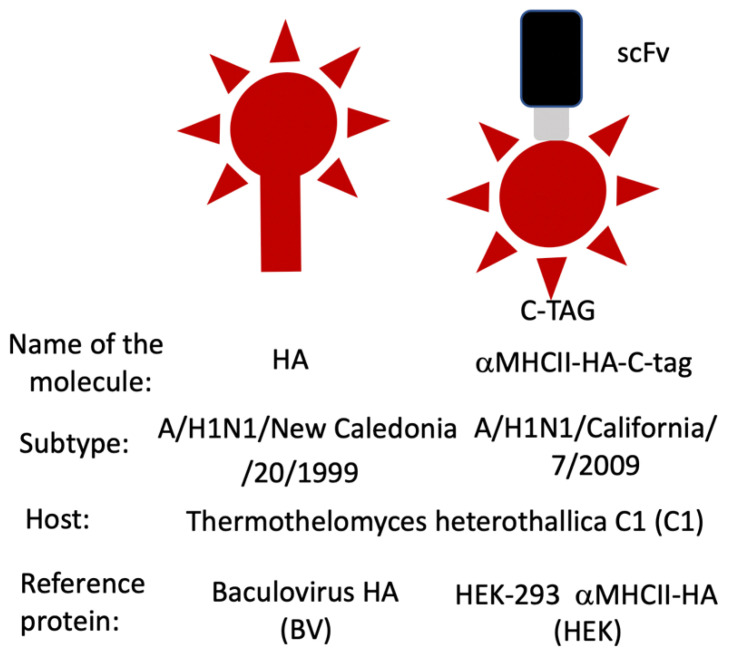
Schematic representation of the two different types of influenza HA immunogens produced in this study in the filamentous fungus *Thermothelomyces heterothallica* C1. The red structure denotes the influenza hemagglutinin (HA). The black box represents the scFv (single-chain variable fragment). The gray box represents the glycine-rich linker connecting the scFv with the HA. C-tag denotes a four amino acid tag placed in the C-terminus of the fusion protein.

Figure 1 shows the types of proteins expressed in C1 in the present study, which are designed to be the immunogens of influenza hemagglutinin (HA) subunit vaccines.

### 2.2. Generation of Strains

*Thermothelomyces heterothallica* (also as *heterothallicus*) [29,30,31], C1 [32] is a thermotolerant haploid ascomycetous fungus. It has been improved using decades of random mutagenesis for improved fermentation properties for the production of secreted cellulase and other enzymes [33,34], giving rise to strain C1, which has received a "generally recognized as safe” (GRAS) status from the FDA [35]. A number of C1 strains have been further engineered using a number of rational strain engineering steps to be suitable for the expression of xenogeneic proteins. For specific details of the strain development technology see [20] and (manuscript in preparation). Furthermore, glycoengineered C1 strains that incorporate mammalian type N-glycans such as G0 and G2 have been produced (manuscript in preparation); however, it is important to note that this work has been performed in C1 strains incorporating Man3-Man9 fungal N-glycans.

The generation of the strain expressing the full-length HA (*New Caledonia/20/99*) is described in [36]. The coding sequences of αMHCII-HA-C-tag (*California/7/2009*) were codon-optimized, placed in the context of an efficient C1 signal sequence, and embedded into an insertion vector that contained appropriate 5′ and 3′ regulatory sequences [37] to drive a constitutive strong expression of the gene of interest, appropriate selectable marker gene cassette, and targeting arms for the integration of the construct into well-defined hot spots of the haploid *Thermothelomyces heterothallica* strain C1 fungal genome. The linearized vectors were introduced into the fungal protoplasts of the production strains using a PEG-mediated transformation process. The transformed protoplasts were grown to colonies on selective medium and optionally genotyped using colony PCR, and phenotyped in medium throughput fermentation in 24-well plates. The selected colonies were then reisolated to ensure homonuclearity, and rescreened. At this stage a Working Cell Bank was established and validated. For details see (manuscript in preparation)**.**

### 2.3. The Upstream Process—Production of Influenza Antigens

The fermentation of the C1 strain producing a full-length HA (*New Caledonia/20/99)* is described in [36]. The strain producing αMHCII-HA-C-tag (*California/7/2009*) was produced by fed-batch fermentation in 0.15- and 1-L scales. Batch phase: The cultures were grown from a 1-mL Working Cell Bank, first in shaken flasks, then in stirred-tank plastic/stainless steel/glass fermenters maintaining a pO_2_ of 20% on 38 °C using an inoculation ratio of 10–20×. The medium contained the carbon and nitrogen sources, minerals, salts, and “vitamins”. The carbon source used was glucose. The nitrogen source was mainly inorganic (in the form of ammonium ion) supplemented with yeast extract as a complex nitrogen. In the shaken flask the acidification was countered by using a high-buffer capacity medium. In the stirred tank the pH was set to 6.8 using ammonium hydroxide feeding. Feeding phase: After the culture reached the desired scale and density, and the culture had consumed the initial glucose provided in the batch fermentation medium, feeding was initiated. Glucose was used as a carbon source. Vitamins and yeast extract were fed to replenish the consumed material. The pH was maintained at pH = 6.8 using ammonium hydroxide feeding. All materials used were of non-animal origin and are readily available as GMP-grade from multiple commercial sources. The fermentations were sampled as needed. For details see (manuscript in preparation).

### 2.4. The Downstream Process—Purification of Influenza Antigens

**Figure 2:**
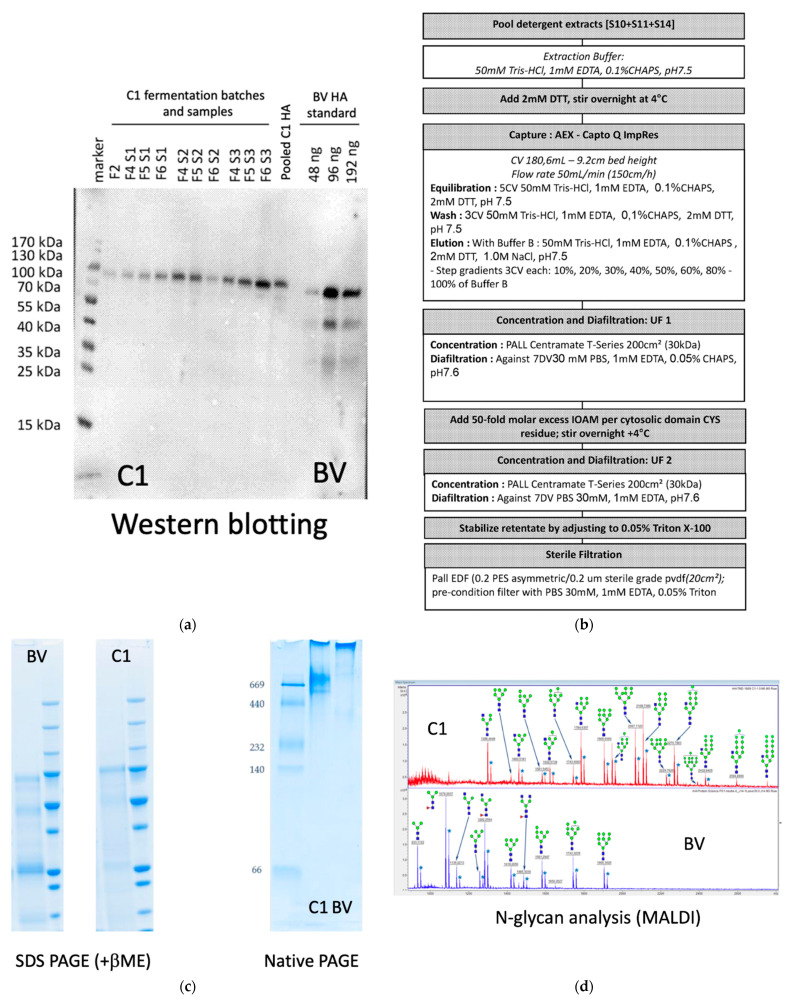
Expression of Hemagglutinin (HA) (*New Caledonia/20/99*) in C1. (**a**) Expression of HA protein in the biomass fraction of the C1 fermentation broth. F2, F4, F5, and F6 are independent fermentation runs, whereas S1, S2, and S3 are different samplings on days 1, 2, and 3. The biomass fraction was collected by centrifugation and assessed by Western blotting. The “Pooled C1 HA” lane contains the purified HA from the pooled fermentation broth, which also was used for immunization. The HA protein was detected by anti-influenza-A/H1N1/HA (*New Caledonia/20/99*) antibody. (**b**) Downstream process for the purification of HA from the cell mass. (**c**) Analysis of the purified HA protein. SDS-PAGE under reducing conditions showed that the sample contains predominantly HA. Native PAGE results show that most of the material is in high molecular form. (**d**) Comparison of the N-glycan forms present on C1-HA (*New Caledonia/20/99*) and BV-HA (*New Caledonia/20/99*). Blue square, green circles, and red triangles denote N-acetyl-glucosamine, mannose, and fucose, respectively.

For the purification of the full-length HA (*New Caledonia/20/99*) the fermentation culture was centrifuged and the pellet containing the biomass was resuspended and extracted with extraction buffer containing 2 mM of DTT as in Figure 2b. The sample was then again centrifuged, and material from the supernatant samples was separated by SDS-PAGE, transferred to PVDF membrane. The hemagglutinin protein was detected by Western blotting as in 2.7. As a control HA (*New Caledonia/20/99*) protein from Baculovirus-based expression system (Protein Biosciences, now Sanofi Pasteur, Lyon, France) was used as in [36]. Figure 2b shows the downstream process for the isolation of hemagglutinin from the cell mass. In the first step the biomass was separated from the supernatant by centrifugation. The biomass fraction was solubilized with the extraction buffer supplemented with 2 mM DTT overnight at 4 °C. The extract was loaded onto Capto^TM^ Q ImpRes (Cytiva, Uppsala, Sweden) anion exchange resin and the column was washed with 3 column volume extraction buffer supplemented with 2 mM DTT. These conditions allowed the binding of the majority of hemagglutinin content of the extract (data not shown). The bound material was eluted as fractions with a stepwise gradient of 3 column volumes of extraction buffer containing increasing amount of NaCl up to a concentration of 1.0 M as detailed in Figure 2b. The fractions containing the bulk of the hemagglutinin were identified by Western blotting, pooled and concentrated in Centramate T-series (Pall, New York, NY, USA), and the buffer changed to PBS containing 1 mM of EDTA and 0.05% CHAPS. Fifty molar excess iodoacetamide (Sigma–Aldrich, Burlington, MA, USA) were added to the sample mainly to prevent the molecules from forming aggregates by cross-linking via the sulfhydryl groups of the intracellular parts. After overnight incubation the samples were further concentrated, and the remaining CHAPS detergent removed by diafiltration. The resulting samples were supplemented with 0.05% Triton X-100 and sterile filtered.

The procedure was also performed in fermentation samples derived from the parental C1 strain. Samples from different stages of this procedure dialyzed against PBS were used as controls (Mock controls 1 and 2).

For the purification of the αMHCII-HA-C-tag (*California/7/2009*) PMSF was added to the final concentration of 1 mM to the supernatant, and the sample was clarified by centrifugation 3 × 20 min at 20,000× *g* at + 4 °C followed by filtration through a 0.45-µM filter. The cleared supernatant was diluted threefold with PBS supplemented with 0.5 M of NaCl. The C-tag affinity purification was performed on 1 mL of CaptureSelect C-tag XL column (Thermo Fisher, Bleiswijk, The Netherlands) equilibrated with 5 column volumes (CV) of PBS supplemented with 0.5 M of NaCl attached to the ÄKTA Start protein purification system (Cytiva, Uppsala, Sweden). After sample loading, the column was washed with 15 CV of PBS supplemented with 0.5 M of NaCl (wash) and eluted with 10 CV of 20 mM of Tris-HCl, 2 M of MgCl_2_, 1mM of EDTA, and pH = 7.5 with fraction volume of 1 mL. The fractions containing the bulk of the hemagglutinin were identified by Western blotting and concentrated.

### 2.5. Control Proteins

A full-length trimeric HA (*New Caledonia/20/99*) produced in a Baculovirus-based expression system was obtained from Protein Sciences (now Sanofi Pasteur, Lyon, France) as research-grade material.

αMHCII-HA (*California/7/2009*) was produced in HEK293E cells. In brief, DNA enabling the secretion of αMHCII-HA (*California/7/2009*) was mixed with polyethylenimine (PEI) (P3143, Sigma–Aldrich, Burlington, MA, USA) in OptiMem (51985-026, Gibco, Thermo Fisher, Bleiswijk, The Netherlands) and incubated for 20 min at room temperature (RT) and added to 293E cells in 5-layered cell culture flasks (734-2457, VWR), and incubated for 48 h at 37 °C and in a humidified atmosphere containing 5% CO_2_. Supernatants were collected, filtered, and purified on a sepharose column containing the monoclonal antibody 29E3 [38] as an affinity ligand. The collected supernatant was passed through this affinity column twice, and the resulting eluate dialyzed against PBS and sterilized through a 0.2-µm Filtropur S filter (23966, Polysciences, Warrington, PA, USA).

### 2.6. Analytical Methods for the Characterization of the Expressed Proteins

Native and polyacrylamide gel electrophoresis (PAGE) was performed as known in the art. HA (*New Caledonia/20/99*) was detected with Ab661189 (Abcam, Cambridge, UK). HA (*California/7/2009*) was detected with the monoclonal antibody 29E3 [38]. C-tag was detected by Capture Select Biotin Anti-C-tag conjugate (Thermo Fisher, Bleiswijk, The Netherlands). Protein concentrations were assessed by densitometric analysis of samples on the blots using control proteins of known concentrations as a reference.

For N-glycan analysis the hemagglutinin samples were denatured and de-N-glycosylated using PNG-aseF (G5166, Sigma–Aldrich, Burlington, MA, USA) as recommended by the manufacturer. The N-glycans were either analyzed by MALDI [36] or labeled with a fluorescent tag, and analyzed with LC-MS using the RapiFluor-MS N-Glycan Kit (Waters, Milford, MA, USA). The assignment of some of the high molecular-weight peaks to exact glycostructures present in HA produced in C1 expression systems is tentative, as no appropriate control N-glycans are commercially available.

### 2.7. Animal Studies

**Figure 3:**
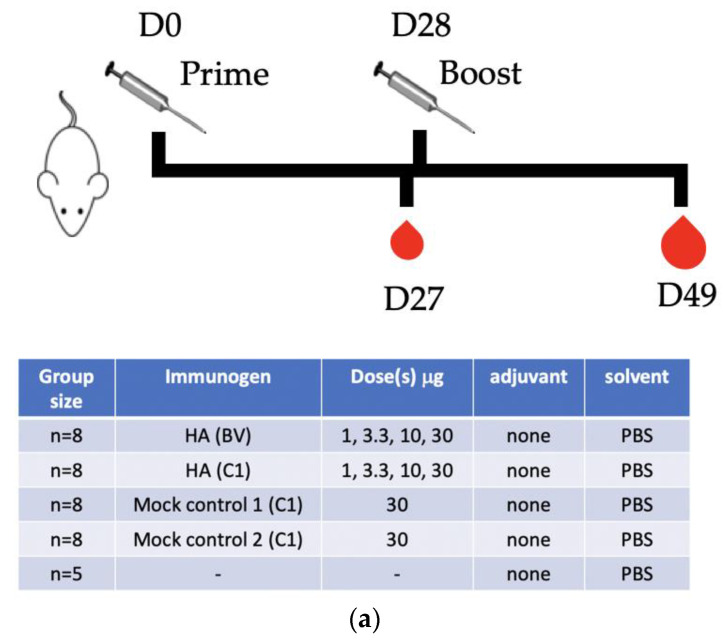

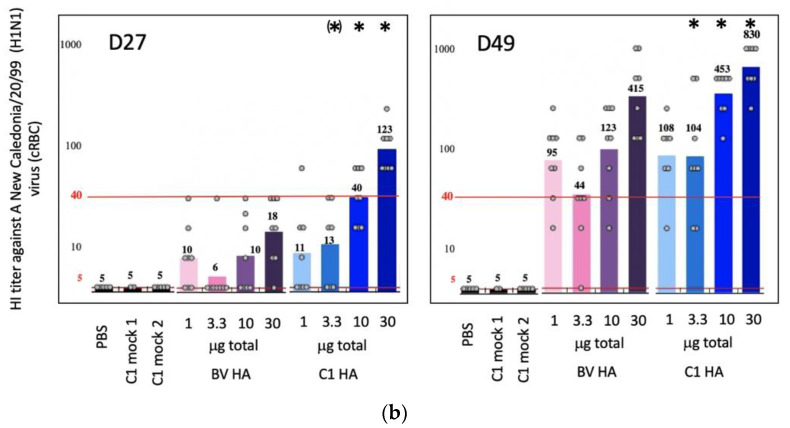
Immunological response of mice to Hemagglutinin (*New Caledonia/20/99*) produced in C1 (C1 HA) and Baculovirus-based expression system (BV HA). (**a**) Setup of the mouse immunological study. (**b**) Assessment of sera from the various study groups for their ability to inhibit hemagglutination as assessed by the Hemagglutination Inhibition (HI) assay. C1 mock 1 and mock 2 are protein extracts from the parental C1 strain not expressing any HA. The HI titer of 5 is set as an arbitrary value for non-responders to expedite statistical analysis. Stars above the bars indicate significant (*p* > 0.05, Student *t*-test) increase in HI titers in mice immunized with the C1 produced HA when compared to mice immunized with equivalent amount of BV HA. Parenthesized asterisk means that care should be taken to evaluate the results of the statistical analysis due to the large fraction of non-responders in at least one of the groups. The HI titer of 40 is shown as an arbitrary threshold for a protective antibody response in human, though its applicability in mice has not been studied in detail.

The assessment of the immunogenicity of the C1-produced (*New Caledonia/20/99*) mice (Figure 3a) was conducted at Sanofi Pasteur. Balb/c By1 mice (Charles River, Cambridge, MA, USA) aged 9 weeks +/− 3 days at day 0 (D0) were used. Mice were kept under SPF conditions in an animal facility meeting L2 biosafety requirements. Mice were fed with granulated food (M20, SDS, Dietex, St. Gratien, France) and tap water ad libitum. The mice were acclimated to their designed housing for 5 days before D0. On D0 the mice received 2 × 50 μL intramuscular injection into the quadriceps. Mice were injected either with PBS (5 animals each), two different batches of C1 mock protein samples that were obtained from non-transformed parental strains, and which have undergone the same purification process as above (5 animals each), or with increasing dosage (1, 3.33, 10, and 30 μg per dose of either C1-produced or Baculovirus expression system produced HA (*New Caledonia/20/99*) (8 animals each)). On D27 the mice were weighed, and blood was sampled from the submandibular vein under isoflurane anesthesia. On D28 the mice again received the same injection as on day 1 (boost). On D49 the mice were again weighed, anesthetized by intraperitoneal administration of 200 μL of Imalgene (1.6 mg of ketamine) and Rompun (0.32 mg xylazine) and exsanguinated from the carotid section. The sera were collected and stored at −20 °C until analysis.

The assessment of the immunogenicity of the C1-produced αMHCII-HA (*California/7/2009*) in mice (Figure 5a) was conducted at the at Oslo University Hospital, Norway. Six-to-eight-week-old female BALB/c mice (Janvier, le Genest-Saint-Isle, France) were housed under minimal disease conditions. Proteins dissolved in saline, or formulated with AS03 (GlaxoSmithKline, GSK, Uxbridge, UK), were injected intramuscularly (i.m.) into each quadriceps. Flublok Quadrivalent (#90686, Sanofi Pasteur, Orlando, FL, USA) referred to shortly as Flublok was used as a control vaccine. Blood samples were collected biweekly from mice by puncture of the saphenous vein, and sera isolated by two successive centrifugations for 5 min at 13,000 rpm. On week 16, groups of anaesthetized mice were infected intranasally with 5 × LD50 of influenza A H1N1 (*California/7/2009*) in 10 µL per nostril. Mice were monitored for weight loss with an endpoint of 20% weight reduction, as required by the Norwegian Food Authority. Mice reaching the >20% weight loss were euthanized by cervical dislocation.

### 2.8. Hemagglutination Inhibition (HI) Assay

All sera were pretreated with the receptor destroying enzyme (Sigma-Aldrich, Burlington, MA, USA), then absorbed on 10% chicken red blood cells (cRBC) (prepared in-house at Sanofi), and finally treated with trypsin. Allantoic fluid containing influenza strain A/H1N1/ (*New Caledonia/20/99*) was diluted to contain 4 hemagglutination unit (HAU) per 50 μL. Fifty μL of this suspension was added to wells V-bottom 96-well plates containing 50 μL of the 2× serial dilution of the antisera (starting dilution 1:10, dilution in Ca^2+^ and Mg^2+^ free PBS (Gibco, Thermo Fisher, Bleiswijk, The Netherlands). The plate was incubated for one hour at room temperature. Fifty μL cRBC (0.5% in PBS) was then added to each well, and the plate was incubated for one hour at room temperature. The hemagglutination was assessed visually as distinct red dot at the bottom of the well vs. pink diffuse halo in the wells. The HI titer is the last dilution that completely inhibited hemagglutination. Each plate contained no antibody, and no A/H1N1/ (*New Caledonia/20/99*) controls, and the results were only deemed valid if the controls behaved as expected.

### 2.9. Sandwich ELISA

Sandwich ELISAs were performed with recombinant HA from influenza A/H1N1/ (*California/7/2009*) (11055-V08H, Sino Biological, Beijing, China) as the coat, blocked with 0.1% BSA in PBS, and incubated overnight at 4 °C with titrated amounts of sera from mice assayed individually. Antibodies were detected with biotinylated anti-IgG (A2429, Sigma Aldrich, Burlington, MA, USA), anti-IgG1 (553599, BD Pharmingen, San Diego, CA, USA), or anti-IgG2a (553502, BD Pharmingen, San Diego, CA, USA), followed by streptavidin alkaline phosphatase (GE Healthcare, Chicago, IL, USA) and phosphatase substrate (P4744-10G, Sigma Aldrich, Burlington, MA, USA), and quantitated with a Tecan reader using the Magellan v5.03 software. Titers were given, defined as the last serum dilution giving an absorbance above background (mean absorbance for NaCl-vaccinated mice plus five times SEM).

## 3. Results

### 3.1. Influenza Hemagglutinin Produced in C1 (C1-HA (New Caledonia/20/99))

Several full-length membrane-linked HA proteins were expressed in C1, including those derived from the pre-2009 pandemic A/H1N1/(*Puerto Rico/8/1934*) and A/H1N1/(*New Caledonia/20/99*), the A/H3N2/ *(Texas/1/1977)* and the 2009 pandemic A/H1N1/(*California/7/2009*) strains, as well as the B *(Florida/4/2006)* strain. All HA subtypes were functionally active as judged by agglutination bioassays (data not shown). Since the best yield and quality was obtained with the HA (*New Caledonia/20/99*) and the same HA antigen was available from a baculovirus-based expression system (BV-HA (*New Caledonia/20/99*)), a method was developed to purify the HA from the C1 host (C1-HA (*New Caledonia/20/99*))). The fermentation process was briefly optimized (Figure 2a), and consequently the yield of HA (*New Caledonia/20/99*), was notably increased to 300–600 mg/L. Western blot analyses of various batches at the end of the fermentation process revealed the presence of a recombinant protein that reacted against an anti-HA (*New Caledonia/20/99*) antibody and whose migration pattern was slightly higher but consistent with the migration pattern of the unprocessed HA0 as observed in the BV-HA (*New Caledonia/20/99*) control (Figure 2a). Please also note that likely due to the absence of extracellular proteases in the C1 system capable of the recognizing the HA1/HA2 junction, a single HA0 protein band was observed in C1-HA (*New Caledonia/20/99*) samples. In contrast, the BV-HA (*New Caledonia/20/99*) shows protein bands with migrations patterns consistent with partial processing of HA0 into HA1 and HA2 products (Figure 2a).

A downstream process was developed to extract the C1-HA (*New Caledonia/20/99*) protein from the cell mass (Figure 2b). The downstream process led to extraction of the target protein with a purity of ca. 80–90% as revealed by denaturing SDS PAGE under reducing conditions (Figure 2c). Native PAGE showed that most of the HA was present as a high molecular weight product consistent with homotrimers of HA0 like the in the control BV-HA (*New Caledonia/20/99*).

Since slight differences in migration of protein products were observed between C1-HA (*New Caledonia/20/99*) and BV-HA (*New Caledonia/20/99*), we investigated the types of N-glycosylation present on these proteins. The C1 system produced HA (*New Caledonia/20/99*) characterized mainly by N-glycan forms Man3-Man9, by optional N-acetyl-glucosaminylation of only one of the two terminal mannoses, and the lack of fucosylated N-glycan species (Figure 2d top). In contrast, the BV-HA (*New Caledonia/20/99*) showed a glycosylation pattern that included N-acetyl-glucosaminylation of both terminal mannoses of the Man3 core as well as fucosylation of some of the N-glycan side chains (Figure 2d bottom). The higher mannose content of the C1-HA (*New Caledonia/20/99*) compared to the BV-HA (*New Caledonia/20/99*) is consistent with the migration pattern observed by Western blot.

### 3.2. C1-HA (New Caledonia/20/99) as an Influenza Vaccine Candidate

To assess the immunogenicity of C1-HA (*New Caledonia/20/99*), a mouse study was conducted (Figure 3a). In brief, mice were immunized using a prime-boost regime 28 days apart with various doses of unadjuvanted C1-HA (*New Caledonia/20/99*), or BV-HA (*New Caledonia/20/99*). PBS and mock protein samples from the parental strains were used as controls (Figure 3b). HI antibodies titers present in mouse sera on days 27 (left panel, post-prime) and 49 (right panel, 21 days post-boost) were assessed. HI antibodies were readily detected after prime immunization in the high dose protein groups (10 and 30 µg dose), but only the group immunized with C1-HA (*New Caledonia/20/99*) had a significant number of animals consistently showing HI titers of ≥40 predictive of protection. This was particularly the case in the group receiving 30 µg of C1-HA (*New Caledonia/20/99*) showing HI titers ≥80. Increased HI responses were observed on samples on day 49, consistent with the boost response. Notably, HI titers after boost were consistently higher in groups immunized with C1-HA (*New Caledonia/20/99*) compared to groups immunized with identical amounts of BV-HA (*New Caledonia/20/99*). Overall, HI titers were statistically higher in the groups immunized with C1-HA (*New Caledonia/20/99*) than in groups immunized with identical amounts of BV-HA (*New Caledonia/20/99*) in the 3.3- to 30-µg dose range. These results suggest that the C1-produced HA is immunogenic and showed an improvement in neutralizing HI responses compared to those derived from the Baculovirus-based expression system.

Importantly, an expected increase of the mean body weight of mice in all study groups was observed, showing that the C1-produced proteins (immunogen samples, mock protein samples) did not have any unexpected toxic effects on the animals in the doses investigated.

### 3.3. A Secreted MHCII-Targeted HA (California/7/2009) Produced in C1

To produce a secreted HA variant that would be easier to purify than the membrane-bound HA and to further improve its immunogenicity, we expressed a fusion protein consisting of the HA1 (*California/7/2009*) region fused to a glycine-rich linker followed by a single chain variable fragment. The fusion construct has been described in detail in [39,40,41]. Previous studies have shown that linking an antigen to scFv targets the antigen to αMHCII receptors thereby improving immunogenicity [39,40,41] and the elicitation of strong neutralizing antibodies in mice and larger animals [42,43,44]. In addition, a C-terminal tag (C-tag) [45] was appended to the C-terminus to expedite purification of C1 αMHCII-HA (*California/7/2009*) C-tag protein.

**Figure 4:**
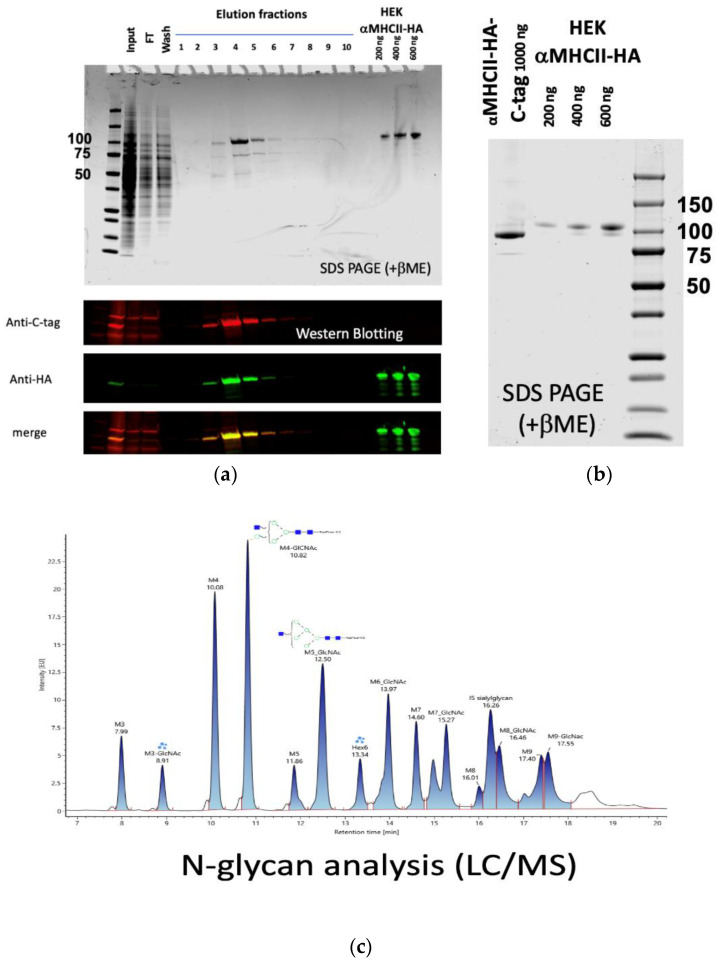
Expression of C1 αMHCII-HA (*California/7/2009*)-C-tag. (**a**) Expression of HA in the supernatant of the C1 fermentation broth and its purification. C1 αMHCII-HA (*California/7/2009*)-C-tag was expressed as a ca. 100 kB protein and assessed by WB using anti-HA (*California/7/2009*) antibody and anti-C-tag antibody. Note that the HEK αMHCII-HA (*California/7/2009*)-reference protein is not C-tagged, and therefore cannot be detected using the anti-C-tag antibody. The fractions containing the bulk of the HA were identified by Western blotting, pooled, and concentrated. (**b**) Purified C1 αMHCII-HA (*California/7/2009*)-C-tag as assessed by SDS-PAGE under reducing conditions compared with the reference protein HEK αMHCII-HA (*California/7/2009*). (**c**) The N-glycan pattern of the purified C1 αMHCII-HA (*California/7/2009*)-C-tag showing the characteristic fungal N-glycosylation profile. This is significantly different from the human N-glycosylation pattern characteristic, which explains the apparent different migration properties of the C1 αMHCII-HA (*California/7/2009*)-C-tag and HEK αMHCII-HA (*California/7/2009*) seen in (**b**), the N-glycan profile of which was not investigate within this study. Note that ‘IS sialylglycan’ is an internal analytical glycan standard. Blue square and green circles denote N-acetyl-glucosamine and mannose, respectively.

Here, the αMHCII-targeted HA1 fragment was expressed in the C1 system (C1 αMHCII-HA (*California/7/2009*) C-tag) and compared to the same product without the four amino acid C-tag produced in human embryonic kidney HEK-293 cells (HEK αMHCII-HA (*California/7/2009*). The recombinant αMHCII-HA-C-tag/*α*MHCII-HA proteins were readily expressed in both systems and could be detected by antibodies directed against HA (*California/7/2009*) and the C-tag (where applicable). The C1 αMHCII-HA (*California/7/2009*) C-tag protein was produced at 300–600 mg/L and it was purified using the C-tag as the affinity ligand (Figure 4b). Analysis of the N-glycan profile present on the C1 αMHCII-HA (*California/7/2009*) C-tag showed the typical C1 N-glycan forms similar to the C1-produced full-length HA (*New Caledonia/20/99*) products.

### 3.4. C1 αMHCII-HA (California/7/2009)-C-Tag as an Influenza Vaccine Candidate

**Figure 5:**
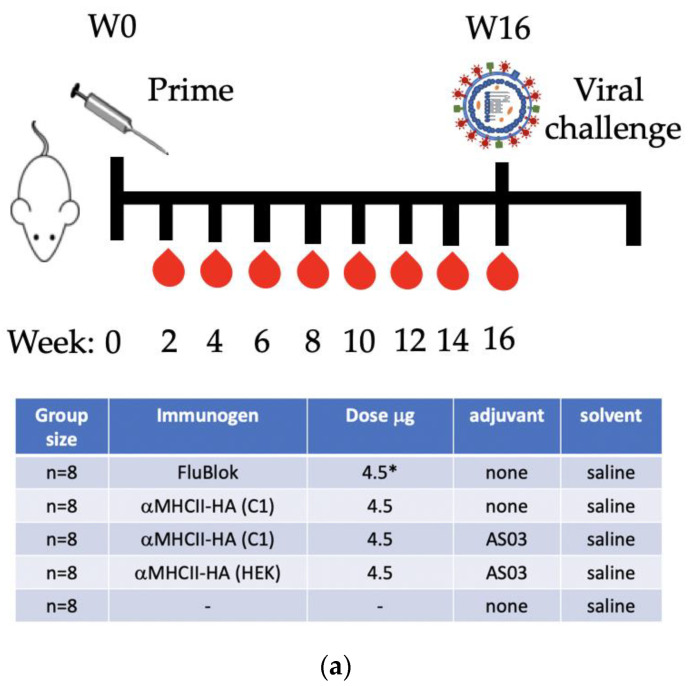

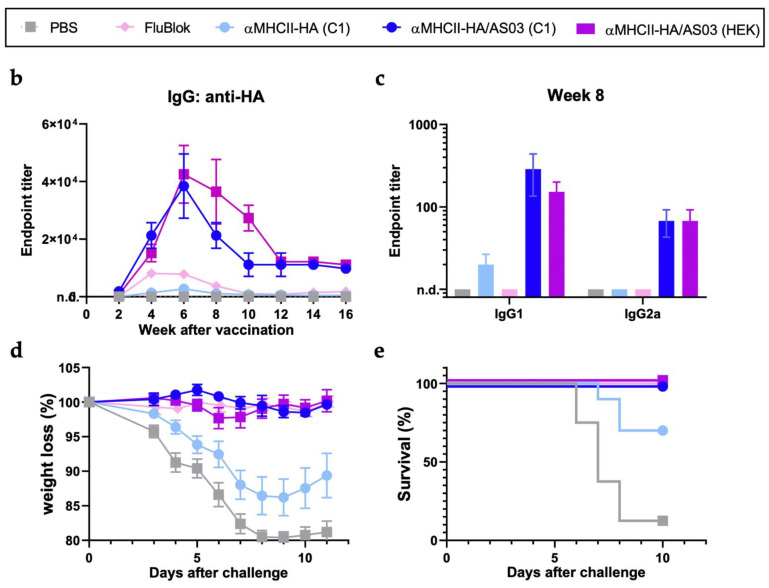
Antibody responses and protection against influenza *A/California/07/2009 (H1N1)* in mice vaccinated with C1 and HEK293E produced immunogens. (**a**) Mice were vaccinated once i.m. with the indicated vaccines, each formulated to contain 4.5 µg of HA specific for A/H1N1/ (*California/7/2009*). Note that Flublok being a quadrivalent vaccine contains HA specific for other subtypes of influenza as well, and immunization with other subtypes may have contributed to the immune response against A/H1N1/ (*California/7/2009*) observed in the experiment. Blood was collected longitudinally, followed by an influenza A/H1N1/ (*California/7/2009*) challenge at week 16. (**b**) Serum IgG responses measured longitudinally in ELISA against recombinant HA (*California/7/2009*) (mean+/− SEM). *p* < 0.05 for C1 and HEK αMHCII-HA/AS03 as compared to NaCl, two-way ANOVA with a Bonferroni post test. By the same test, Flublok was significantly different from NaCl at weeks 4 and 6. (**c**) IgG1 and IgG2a responses in sera collected at 8 weeks post a single vaccination. * *p* < 0.05 as compared to NaCl, Mann–Whitney. (**d**,**e**) Mice were challenged with a 5 × LD50 dose of influenza A/H1N1/ (*California/7/2009*), and weight monitored (**d**). * *p* <0.05 as compared to αMHCII-HA. (**e**) Survival was assessed in relation to 20% weight loss as the humane endpoint. * *p* <0.05, Mantel–Cox test.

In order to evaluate immunogenicity, mice (*n* = 8/group) were vaccinated once with αMHCII-HA produced in either the C1 or HEK293E expression systems (Figure 5a) and compared to responses elicited by the licensed quadrivalent vaccine Flublok produced in baculovirus that contains the same HA (*California/7/2009*) [21]. C1 αMHCII-HA (*California/7/2009*)-C-tag and HEK αMHCII-HA (*California/7/2009*) proteins were formulated to contain the equivalent of 4.5 μg of HA in absence or presence of the AS03 adjuvant. Sera from the vaccinated mice collected longitudinally every 2 weeks from the second week post-vaccination until week 16 were evaluated for anti-HA (*California/7/2009*) IgG responses by ELISA. Strong and comparable anti-HA (*California/7/2009*) IgG responses were observed in mice vaccinated with the C1-derived and HEK293-derived αMHCII-HA-C-tag or αMHCII-HA proteins, respectively, when formulated with AS03 adjuvant. Anti-IgG levels were significantly higher than those elicited by Flublok, the latter being above the baseline only in serum samples from weeks 4 and 6. In contrast, sera from mice vaccinated with the C1 αMHCII-HA (*California/7/2009*)-C-tag without adjuvant showed weak responses only detected on samples from week 6 post-vaccination. These trends were also observed when evaluating the IgG1 and IgG2a subclasses in sera from 8 weeks post-vaccination (Figure 5c). Both adjuvanted αMHCII-HA formulations elicited strong IgG1 and IgG2a responses, whereas samples from mice receiving either Flublok or non-adjuvanted αMHCII-HA showed detectable IgG1 responses but negligible IgG2 responses.

At week 16 post-vaccination, mice received intranasally a lethal dose (5 × LD50) of the homologous influenza A/H1N1/ (*California/7/2009*) strain to assess the protective efficacy of the different vaccines (Figure 5d). Mice vaccinated with the adjuvanted αMHCII-HA formulations or Flublok showed no clinical signs, no body weight loss, and were fully protected. Interestingly, mice that received the C1-derived non-adjuvanted αMHCII-HA lost weight, but 5 out of 8 animals recovered and survived, suggesting that even non-adjuvanted formulation conferred some protection. As expected, the mock-vaccinated-challenged mice experienced the most rapid weight loss and 7/8 were humanely euthanized due to reaching humane endpoints (Figure 5e).

## 4. Discussion

In this report we provide evidence of the potential of the C1 system as an alternative to produce protein-based subunit influenza vaccines. This and prior studies suggest that the C1 expression system produces higher amounts of proteins per volume than other common expression systems such as the insect BV system or mammalian HEK-293E expression system. In nature, the *Thermothelomyces heterothallica* fungus survives by secreting lignocellulose degrading enzymes that decompose lignocellulosic materials to oligomers and monomers, which are retrieved to support growth of the organism [46]. As the growth and the survival of the species is directly coupled to the secretion of proteins, the wild-type *Thermothelomyces heterothallica* seemed an ideal host to be developed to an expression system for producing secreted recombinant proteins. The wild-type C1 strain was improved for two decades in the microbial industrial space to produce and secrete up to more than 100 g/L of lignocellulose degrading enzymes using low-cost microbial fermentation media. To convert this potential to express xenogeneic proteins relevant for the pharmaceutical industry the strain was further modified by knocking out more than a dozen protease genes and knocking down or eliminating the expression of C1 intrinsic secreted proteins. The result is a C1 strain that shows improved stability, yield, and purity of multiple types of expressed xenogeneic proteins, such as immunogens, monoclonal antibodies and other therapeutic proteins.

The full-length HA (*New Caledonia/20/99*) protein produced in C1 were most likely expressed as HA0 products based on migration patterns under denaturing and native PAGE conditions. As mentioned above, the C1 strain has been systematically engineered to produce fewer proteases, and this may explain the lack of processing of HA0 to HA1 and HA2 polypeptides. As expected, the HA antigens from the C1 system show N-glycosylation profiles that are different from those observed in similar antigens produced in mammalian cells. For influenza, altering glycosylation profiles either display or mask antigenic regions. Changes in glycosylation patterns is an integral part of influenza virus antigen evolution [47,48]. As such, efficient vaccine development should consider the glycan structure of the produced immunogens. Further studies are needed to evaluate the potential adjuvant effect gained by nonhuman N-glycosylation, but also to see if the different glycosylation profiles enabled by C1 glycoengineered strains will stimulate immune responses that differ in their ability to broadly bind antigenically drifted strains of influenza. Since several C1 glycoengineered strains are available, such as G0 and G2 C1 strains, future studies are warranted. Interestingly, the non-glycoengineered C1-HA (*New Caledonia/20/99*) elicited stronger HI responses than BV-HA (*New Caledonia/20/99*). Whether this effect is attributable to different N-glycosylation profiles, the dominant presence of the HA0 form in C1-HA (*New Caledonia/20/99*), or any other aspect (e.g., different purity profile, including product-related and host-related impurities) needs additional studies beyond the scope of this report.

While C1-HA (*New Caledonia/20/99*) showed promising immunogenic properties, the purification of the protein is cumbersome, both the yields are low (ca. 50%) and final purities (80–90%) are mediocre. The process is also challenging to scale up to industrial scales. We have tried to express truncations of HA (*New Caledonia/20/99*), as a secreted protein, but these efforts were not successful. Expressing truncated HA with fusion partners allowed, however, the functional expression of HA and many other proteins. Previous studies have shown that antigens engineered with scFv can more efficiently target MHCII receptors in antigen presenting cells. We therefore expressed an MHCII targeted HA (*California/7/2009*) fusion protein, which was efficiently secreted to the fermentation broth. With an introduced tag the secreted protein could be purified to >95% purity with a yield of >90% in a single step. When αMHCII-HA (*California/7/2009*) from either the C1 or the HEK 293 expression system were formulated with the AS03 adjuvant, strong anti-HA IgG responses were readily detected in which both IgG1 and IgG2a responses were significantly higher compared to samples from Flublok-vaccinated animals and non-adjuvanted C1-derived αMHCII-HA (*California/7/2009*). Furthermore, all immunized animals survived a lethal viral challenge, showing that the produced C1 αMHCII-HA (*California/7/2009*) is efficient immunogen as the HEK αMHCII-HA(*California/7/2009*).

We (Dyadic International Inc.) conducted the two different animal studies shown here with different partners (Sanofi, University of Oslo) in two different sites. The goals of our partners of the two studies were different, which were mirrored in the different setups of the animal studies. In the study shown in Figure 2 and Figure 3, we and our partners were mainly interested if unadjuvanted antigens produced in C1 can raise a protective immune response. In the study shown in Figure 4 and Figure 5 we and our partners were mainly interested if a single immunization can be protective, and therefore adjuvants were part of the experimental design. Note that the influenza subtypes of the H1N1 HA used in the two studies were different (*New Caledonia/20/99* vs. *California/7/2009*) as were the production hosts of the control proteins (Baculovirus-based vs. mammalian expression system). All this would make direct side-by-side comparison of the two studies speculative at this point. However, in both experimental setups the C1-produced immunogens performed identically or better in terms of immunogenicity than the controls produced in prototypic expression systems.

The influenza HA shown here were not the only antigens that were successfully expressed in C1. Rift valley fever virus (RVFV) and Schmallenberg virus (SBV) viral antigens have been expressed at greater than 1.5 g/L in five days in the framework of the Zoonosis Anticipation Preparedness Initiative (ZAPI) [49] and C1 strains have been used to produce the SARS-CoV-2 receptor binding domain (RBD) at greater than 2 g/L in five days [50]. Additionally, C1 strains have also been rapidly developed that express the Alpha, Beta, Gamma, and Delta variant RBDs, the SARS-CoV-2 full spike protein, as well as an Fc-RBD. The RVFV, SBV, and the SARS-CoV-2 RBD antigen have been shown to elicit a strong immune response in animals, and where tested, this immune response was protective upon viral challenge [49]. This shows that the C1 platform can not only be utilized for production of influenza vaccines, but also to help counter a pandemic caused by other types of disease agents.

The C1 technology allows for the quick testing of expression of viral proteins, domains of proteins as non-tagged or tagged variants, or more complex engineered proteins such as ones that may be used for in vivo or in vitro assembly including nanoparticles or virus-like particles [49]. In approximately two months one can generate stable C1 strains using site specific single-copy integration of transgenes to hotspots. The resulting strains can then be used to ferment the antigen of interest and assess its expression level, which can often be much higher than in conventional expression hosts. The antigens then can be rapidly purified and tested in vitro and in animal studies. While animal studies are proceeding, the purification of the immunogen can be further optimized, appropriate analytical methods can be standardized, and initial studies on formulation and stability can be performed.

The current SARS-CoV-2 pandemic clearly shows the importance of achieving protective immunity in the total human population. The concept that a wealthier subpopulation would be immunized with an expensive vaccine is a dangerous one. Escape mutants may emerge especially during pandemics, when the virus adapts to its new human host. Therefore, emphasis should be placed on technologies that allow for fast and low-cost mass production of highly pure stable antigens that can be readily manufactured regionally or locally, that can be used to formulate safe, effective, and affordable vaccines in the quantities needed for a global population. We and a growing number of scientists believe that this is one of the ways a pandemic can be better controlled.

Additional effort should be made to describe the viruses present in human [51,52] and in our environment. The genetic information obtained about viruses can then be used to study which plug-and-play technologies work best with each disease agent. We need to build a knowledge base to counter diseases outside periods of a pandemic that result in a continuous impetus for vaccine platform development. The veterinary industry would surely also benefit because many of our diseases are zoonotic [53,54,55].

To date there has not been any attempt to produce any of the C1 influenza antigens under cGMP. For any novel expression system, the progression from the non-regulated R&D phase to a highly regulated cGMP phase is a steep learning curve. It is therefore important to note that the industrial scale-up under cGMP has been successfully carried out with the C1 produced SARS-CoV-2 RBD.

While no adverse events have been observed in the animal studies described above, no detailed toxicology studies have been conducted with any of the antigens produced in this study. It is therefore worth mentioning that a C1-produced SARS-CoV-2 RBD was not associated with major systemic adverse effects, and it was considered safe in a Toxicology Vaccination Study following four repeated vaccination sessions by intramuscular (i.m.) injections at an interval of 1 week to male and female New Zealand white (NZW) rabbits.

The described C1 technology allows for rapid production of billions of doses of antigens in an efficient, and presumably affordable manner. For example using the >2 g/L productivity (in five (5) days) achieved with the C1-produced SARS-CoV-2 RBD antigen after three months of C1 strain development and up-stream fermentation process improvements, still during the first wave of SARS-CoV-2 pandemic (in Europe) >560 kg of the SARS-CoV-2 RBD immunogen could have been produced (prior to downstream processing) within three (3) months using ten (10) runs with a single 50,000 L standard microbial bioreactor (such as used to produce recombinant insulin) (assuming 80% volume of the fermenter is used, and 70% of the volume is fermentation supernatant containing the product of interest. Taking a conservative 50% estimate for the yield of the downstream process (DSP), >280 kg of the protein could have been purified, assuming that upscaling of the DSP process to those amounts is feasible.

With 25 μg used for a dose in human (extrapolated from the animal studies), this would have translated into 10+ billion doses, sufficient for the immunization of the entire human population (once). Note, that with C1’s productivity and scalability, dose sparing, i.e., utilizing technologies that require smaller doses, is not necessarily required. While more advanced vaccine technologies relying on protein complexes may require less protein per dose, they are usually much slower to develop mainly due to steeply increasing analytical challenges, during a pandemic the simplest and most efficient vaccine manufacturing process may be the fastest and most effective solution for a global population, especially when it comes to middle- and low-income countries.

## 5. Conclusions

The results clearly show the strong potential of *Thermothelomyces heterothallica* C1 strains to produce recombinant proteins, including glycoproteins, to be used as components of vaccines and therapeutics in the pharmaceutical and veterinary industries. The technology described here, while not fully mature, may provide an avenue for quickly reacting to antigenic shifts and drifts even within an influenza season, as the technology is fast to develop, relatively easy to manufacture and is highly scalable.

The SARS-CoV-2 pandemic has clearly shown that our past approaches to counter pandemics did not work optimally. If protein-based vaccine platforms, such as the C1 expression system described here are to remain a viable option as a countermeasure against future pandemics, the developers of the expression hosts, downstream processes and vaccine technologies must all act in coordination to develop plug-and-play vaccine production platforms that can be rapidly deployed against a new disease agent in a short time. This means that a sufficient number of doses can be manufactured within one year or less of the emergence of the disease agent. The vaccines must be produced affordably and in sufficient quantity to immunize the entire human population at a cost that middle- and low-income countries can also afford, in our mutually shared goal of achieving protective immunity in the entire human population.

## 6. Patents

Part of the current work is described in WO/2019/038623 A1 “Production of Flu Vaccine in *Myceliophthora thermophila*”.

## Data Availability

Detailed information on the strains, strain engineering methods (Section 2.2), fermentation (Section 2.3), and downstream processing (Section 2.4) that comprise the know-how of Dyadic International Inc. may be obtained under a Research License. All data supporting the conclusions of this study are contained within the article or in patents cited, except where it is stated that data is not shown.

## References

[1] Girard M.P., Cherian T., Pervikov Y., Kieny M.P. (2005). A Review of Vaccine Research and Development: Human Acute Respiratory Infections. Vaccine.

[2] Iuliano A.D., Roguski K.M., Chang H.H., Muscatello D.J., Palekar R., Tempia S., Cohen C., Gran J.M., Schanzer D., Cowling B.J. (2018). Estimates of Global Seasonal Influenza-Associated Respiratory Mortality: A Modelling Study. Lancet.

[3] Fields B.N., Knipe D.M., Howley P.M. (2013). Fields Virology.

[4] Bullard B.L., Weaver E.A. (2021). Strategies Targeting Hemagglutinin as a Universal Influenza Vaccine. Vaccines.

[5] Segaloff H., Melidou A., Adlhoch C., Pereyaslov D., Robesyn E., Penttinen P., Olsen S.J. (2019). Who European Region And The European Influenza Surveillance Network Co-Circulation of Influenza A(H_1_N_1_)Pdm09 and Influenza A(H_3_N_2_) Viruses, World Health Organization (WHO) European Region, October 2018 to February 2019. Eurosurveillance.

[6] Bouvier N.M., Palese P. (2008). The Biology of Influenza Viruses. Vaccine.

[7] Westgeest K.B., Russell C.A., Lin X., Spronken M.I.J., Bestebroer T.M., Bahl J., van Beek R., Skepner E., Halpin R.A., de Jong J.C. (2014). Genomewide Analysis of Reassortment and Evolution of Human Influenza A(H_3_N_2_) Viruses Circulating between 1968 and 2011. J. Virol..

[8] Webster R.G., Bean W.J., Gorman O.T., Chambers T.M., Kawaoka Y. (1992). Evolution and Ecology of Influenza A Viruses. Microbiol. Rev..

[9] Saunders-Hastings P.R., Krewski D. (2016). Reviewing the History of Pandemic Influenza: Understanding Patterns of Emergence and Transmission. Pathogens.

[10] Rota P.A., Wallis T.R., Harmon M.W., Rota J.S., Kendal A.P., Nerome K. (1990). Cocirculation of Two Distinct Evolutionary Lineages of Influenza Type B Virus since 1983. Virology.

[11] Goto H., Kawaoka Y. (1998). A Novel Mechanism for the Acquisition of Virulence by a Human Influenza A Virus. Proc. Natl. Acad. Sci. USA.

[12] Doms R.W., Helenius A. (1986). Quaternary Structure of Influenza Virus Hemagglutinin after Acid Treatment. J. Virol..

[13] Skehel J.J., Wiley D.C. (2000). Receptor Binding and Membrane Fusion in Virus Entry: The Influenza Hemagglutinin. Annu. Rev. Biochem..

[14] Gamblin S.J., Haire L.F., Russell R.J., Stevens D.J., Xiao B., Ha Y., Vasisht N., Steinhauer D.A., Daniels R.S., Elliot A. (2004). The Structure and Receptor Binding Properties of the 1918 Influenza Hemagglutinin. Science.

[15] Krammer F. (2019). The Human Antibody Response to Influenza A Virus Infection and Vaccination. Nat. Rev. Immunol..

[16] Lee S., Ryu J.-H. (2021). Influenza Viruses: Innate Immunity and MRNA Vaccines. Front. Immunol..

[17] Schild G.C., Oxford J.S., Dowdle W.R., Coleman M., Pereira M.S., Chakraverty P. (1974). Antigenic Variation in Current Influenza A Viruses: Evidence for a High Frequency of Antigenic “drift” for the Hong Kong Virus. Bull. World Health Organ..

[18] Ray R., Dos Santos G., Buck P.O., Claeys C., Matias G., Innis B.L., Bekkat-Berkani R. (2017). A Review of the Value of Quadrivalent Influenza Vaccines and Their Potential Contribution to Influenza Control. Hum. Vaccines Immunother..

[19] WHO Influenza (Seasonal). https://www.who.int/news-room/fact-sheets/detail/influenza-.

[20] Pérez Rubio A., Eiros J.M. (2018). Cell Culture-Derived Flu Vaccine: Present and Future. Hum. Vaccines Immunother..

[21] Cox M.M.J., Izikson R., Post P., Dunkle L. (2015). Safety, Efficacy, and Immunogenicity of Flublok in the Prevention of Seasonal Influenza in Adults. Ther. Adv. Vaccines.

[22] Zhang C., Maruggi G., Shan H., Li J. (2019). Advances in mRNA Vaccines for Infectious Diseases. Front. Immunol..

[23] Wang C., Horby P.W., Hayden F.G., Gao G.F. (2020). A Novel Coronavirus Outbreak of Global Health Concern. Lancet.

[24] Chams N., Chams S., Badran R., Shams A., Araji A., Raad M., Mukhopadhyay S., Stroberg E., Duval E.J., Barton L.M. (2020). COVID-19: A Multidisciplinary Review. Front. Public Health.

[25] Polack F.P., Thomas S.J., Kitchin N., Absalon J., Gurtman A., Lockhart S., Perez J.L., Pérez Marc G., Moreira E.D., Zerbini C. (2020). Safety and Efficacy of the BNT162b2 mRNA Covid-19 Vaccine. N. Engl. J. Med..

[26] Baden L.R., El Sahly H.M., Essink B., Kotloff K., Frey S., Novak R., Diemert D., Spector S.A., Rouphael N., Creech C.B. (2021). Efficacy and Safety of the MRNA-1273 SARS-CoV-2 Vaccine. N. Engl. J. Med..

[27] Vu M.N., Kelly H.G., Kent S.J., Wheatley A.K. (2021). Current and Future Nanoparticle Vaccines for COVID-19. EBioMedicine.

[28] de Oliveira Daian e Silva D.S., da Fonseca F.G. (2021). The Rise of Vectored Vaccines: A Legacy of the COVID-19 Global Crisis. Vaccines.

[29] Singh B. (2016). Myceliophthora Thermophila Syn. Sporotrichum Thermophile: A Thermophilic Mould of Biotechnological Potential. Crit. Rev. Biotechnol..

[30] Marin-Felix Y., Stchigel A.M., Miller A.N., Guarro J., Cano-Lira J.F. (2015). A Re-Evaluation of the Genus Myceliophthora (Sordariales, Ascomycota): Its Segregation into Four Genera and Description of *Corynascus Fumimontanus* Sp. Nov. Mycologia.

[31] Berka R.M., Grigoriev I.V., Otillar R., Salamov A., Grimwood J., Reid I., Ishmael N., John T., Darmond C., Moisan M.-C. (2011). Comparative Genomic Analysis of the Thermophilic Biomass-Degrading Fungi Myceliophthora Thermophila and Thielavia Terrestris. Nat. Biotechnol..

[32] Visser H., Joosten V., Punt P.J., Gusakov A.V., Olson P.T., Joosten R., Bartels J., Visser J., Sinitsyn A.P., Emalfarb M.A. (2011). RESEARCH: Development of a Mature Fungal Technology and Production Platform for Industrial Enzymes Based on a *Myceliophthora Thermophila* Isolate, Previously Known as *Chrysosporium Lucknowense* C1. Ind. Biotechnol..

[33] Gusakov A.V., Salanovich T.N., Antonov A.I., Ustinov B.B., Okunev O.N., Burlingame R., Emalfarb M., Baez M., Sinitsyn A.P. (2007). Design of Highly Efficient Cellulase Mixtures for Enzymatic Hydrolysis of Cellulose. Biotechnol. Bioeng..

[34] Li J., Lin L., Sun T., Xu J., Ji J., Liu Q., Tian C. (2020). Direct Production of Commodity Chemicals from Lignocellulose Using Myceliophthora Thermophila. Metab. Eng..

[35] GRN, No. 292 Cellulase Enzyme Preparation Derived from a Genetically Modified Strain of *Myceliophthora thermophila*. https://www.cfsanappsexternal.fda.gov/scripts/fdcc/?set=GRASNotices&id=292&sort=GRN_No&order=DESC&startrow=1&type=basic&search=Myceliophthora.

[36] Emalfarb M., Verwoerd T.C., Alfenito M.R., Baer M., Legastelois I., Kazek M.-P., Bernard M.-C., Dubayle J., Kensinger R. (2020). Production of Flu Vaccine in *Myceliophthora thermophila*. U.S. Patent.

[37] Emalfarb M.A., Punt P.J., van Zeijl C.M.J. (2011). Expression-regulating sequences and expression products in the field of filamentous fungi. U.S. Patent.

[38] Manicassamy B., Medina R.A., Hai R., Tsibane T., Stertz S., Nistal-Villán E., Palese P., Basler C.F., García-Sastre A. (2010). Protection of Mice against Lethal Challenge with 2009 H1N1 Influenza A Virus by 1918-like and Classical Swine H1N1 Based Vaccines. PLoS Pathog..

[39] Kawamura H., Berzofsky J.A. (1986). Enhancement of Antigenic Potency in Vitro and Immunogenicity In Vivo by Coupling the Antigen to Anti-Immunoglobulin. J. Immunol..

[40] Grodeland G., Fredriksen A.B., Løset G.Å., Vikse E., Fugger L., Bogen B. (2016). Antigen Targeting to Human HLA Class II Molecules Increases Efficacy of DNA Vaccination. J. Immunol..

[41] Biragyn A., Belyakov I.M., Chow Y.-H., Dimitrov D.S., Berzofsky J.A., Kwak L.W. (2002). DNA Vaccines Encoding Human Immunodeficiency Virus-1 Glycoprotein 120 Fusions with Proinflammatory Chemoattractants Induce Systemic and Mucosal Immune Responses. Blood.

[42] Fredriksen A.B., Sandlie I., Bogen B. (2006). DNA Vaccines Increase Immunogenicity of Idiotypic Tumor Antigen by Targeting Novel Fusion Proteins to Antigen-Presenting Cells. Mol. Ther..

[43] Carayanniotis G., Barber B.H. (1987). Adjuvant-Free IgG Responses Induced with Antigen Coupled to Antibodies against Class II MHC. Nature.

[44] Grødeland G., Fossum E., Bogen B. (2015). Polarizing T and B Cell Responses by APC-Targeted Subunit Vaccines. Front. Immunol..

[45] Jin J., Hjerrild K.A., Silk S.E., Brown R.E., Labbé G.M., Marshall J.M., Wright K.E., Bezemer S., Clemmensen S.B., Biswas S. (2017). Accelerating the Clinical Development of Protein-Based Vaccines for Malaria by Efficient Purification Using a Four Amino Acid C-Terminal ‘C-Tag’. Int. J. Parasitol..

[46] van den Brink J., van Muiswinkel G.C.J., Theelen B., Hinz S.W.A., de Vries R.P. (2013). Efficient Plant Biomass Degradation by Thermophilic Fungus Myceliophthora Heterothallica. Appl. Environ. Microbiol..

[47] Allen J.D., Ross T.M. (2018). H3N2 Influenza Viruses in Humans: Viral Mechanisms, Evolution, and Evaluation. Hum. Vaccines Immunother..

[48] Chang D., Zaia J. (2019). Why Glycosylation Matters in Building a Better Flu Vaccine. Mol. Cell. Proteom..

[49] Aebischer A., Wernike K., König P., Franzke K., Wichgers Schreur P.J., Kortekaas J., Vitikainen M., Wiebe M., Saloheimo M., Tchelet R. (2021). Development of a Modular Vaccine Platform for Multimeric Antigen Display Using an Orthobunyavirus Model. Vaccines.

[50] Espinosa L.A., Ramos Y., Andújar I., Torres E.O., Cabrera G., Martín A., Roche D., Chinea G., Becquet M., González I. (2021). In-Solution Buffer-Free Digestion Allows Full-Sequence Coverage and Complete Characterization of Post-Translational Modifications of the Receptor-Binding Domain of SARS-CoV-2 in a Single ESI–MS Spectrum. Anal. Bioanal. Chem..

[51] Wang L.-F. (2011). Discovering Novel Zoonotic Viruses. WANG, Lin-Fa. Discovering novel zoonotic viruses. N. S. W. Public Health Bull..

[52] Zárate S., Taboada B., Yocupicio-Monroy M., Arias C.F. (2017). Human Virome. Arch. Med. Res..

[53] Meslin F.X., Stöhr K., Heymann D. (2000). Public Health Implications of Emerging Zoonoses. Rev. Sci. Tech..

[54] Wang L.-F., Crameri G. (2014). Emerging Zoonotic Viral Diseases. Rev. Sci. Tech..

[55] Hassell J.M., Begon M., Ward M.J., Fèvre E.M. (2017). Urbanization and Disease Emergence: Dynamics at the Wildlife-Livestock-Human Interface. Trends Ecol. Evol..

